# Epidemiological profile and effectiveness of immediate postpartum contraception in Brazilian women

**DOI:** 10.3389/fgwh.2023.1052224

**Published:** 2023-08-10

**Authors:** Marcelo Luis Steiner, Julia Lorenzini Mendes, Rodolfo Strufaldi, Monica Carneiro, Silvana Aparecida Giovanelli, Mariliza Henrique da Silva

**Affiliations:** ^1^ Department of Gynecology and Obstetrics, Faculdade de Medicina do ABC, Santo André, Brazil; ^2^ Department of Gynecology and Obstetrics, Hospital Municipal Universitário de São Bernardo, São Bernardo do Campo, Brazil

**Keywords:** copper intrauterine devices, long-term reversible contraception, postpartum period, female contraceptive devices, immediate postpartum contraception

## Abstract

**Purpose:**

To determine the epidemiological profile of women who inserted copper intrauterine device (Cu-IUD), subdermal etonogestrel implant (ENG), tubal ligation (TL), depot medroxyprogesterone acetate (DMPA) or did not choose a contraceptive method (NCM) in the immediate postpartum period and compare the contraceptive effectiveness of Cu-IUD and DPMA with non-MAC.

**Methods:**

We analyzed the epidemiological profile of women who inserted copper intrauterine device (Cu-IUD), subdermal etonogestrel implant (ENG), tubal ligation (TL), depot medroxyprogesterone acetate (DMPA) or did not choose a contraceptive method (NCM) in the immediate postpartum. The data was collected by electronic medical records of postpartum women assisted at the University Hospital of São Bernardo do Campo (HMU-SBC) from January 2016 to December 2020. Also, we compared the contraceptive effectiveness of Cu-IUD and DPMA with non-MAC by identifying women who returned for second delivery during the study period and analyzing the contraceptive method chosen in the first hospitalization. Then the pregnancies interval and the sociodemographic characteristics were analyzed according to contraceptive method type.

**Results:**

Data from 20,896 women were collected, of which 8,183 (39%) opted for Cu-IUD, 559 (2.5%) DPMA, and 10,989 (52.5%) chose not to use contraception at the time of hospital discharge. When comparing these groups, women in the DPMA were younger (26.5 ± 7.3, *p* < 0.05), and NCM showed women with a lower number of pregnancies (2.2 ± 1.3, *p* < 0.05). Subjects in the TL group (4.6%) had the higher number of pregnancies (3.8 ± 1.2, *p* < 0.05), and ENG group, the highest number of miscarriages (1.6 ± 1.3, *p* < 0.05). Of those women who returned pregnant, 5.5% belonged to the DPMA group, 6% to the NCM group, and 2.3% to the Cu-IUD.

**Conclusions:**

Women who opted for Cu-IUD insertion were younger, had more pregnancies and vaginal delivery when compared to those who did not choose a method. Of those women who returned, the minority opted for Cu-IUD compared to those that opted for DPMA or no method.

## 1. Introduction

Contraceptive methods play a central role in sexual and reproductive planning, enabling the couple to decide on their pregnancy planning freely and responsibly (1). However, despite the ample supply of methods, about half of pregnancies worldwide are unplanned (2).

Unplanned pregnancy is associated with a higher risk of perinatal complications; some examples are: low birth weight, preterm labor and preeclampsia (3). Women with unintended pregnancies are more vulnerable to developing suicidal ideation, depression and poor nutrition during pregnancy. Also, the risk of physical and psychological violence, unstable family relationships, miscarriage and having low birth weight infants are bigger (22, 23). Furthermore, the risks are more significant if these pregnancies occur within less than eighteen months, with a 61% low birth weight risk and 40% premature birth (4).

The immediate postpartum period can be an opportune time to start a contraceptive method, as pregnant women are usually interested in preventing a new pregnancy and the hospital environment offers a facilitating situation for both doctor and patient (5). The hospital environment is favourable for starting a contraceptive method since the woman is already hospitalized, that is, it is an opportunity for doctors to provide assistance and guarantee guidance on family planning; and also for the patient, who does not need to be hospitalized again to undergo the procedure.

Globally, contraceptive prevalence among married or cohabiting women of reproductive age increased from 55% to 63% over 21 years (1990–2011) (13). In low-income countries, 36% of women who are married or in a stable union use contraceptive methods, while in high-income countries it is twice as high, 66%. Some reasons for this divergence could be lack of knowledge among women, medical dependency for information and provider bias towards permanent contraception (12).

Long-acting reversible contraceptives (LARCs), such as intrauterine devices and hormonal implants, are good options for immediate postpartum contraception (6). Besides having high satisfaction rates, continued use, and high efficacy (8, 11), the WHO has released them for use within the first 48 h after delivery, provided the woman has no contraindications and chooses to leave the maternity hospital with a contraceptive method (6).

A viable option of LARCs for use in the public health system in Brazil is the Copper Intrauterine Device (Cu-IUD) since they are available in the public health network, have high efficacy, have few side effects, and low cost (1). The main concern of its insertion in the immediate postpartum period is expulsion, whose rates can vary from 0% to 13% (5, 6), tend to be higher than in other elective insertion situations (6, 7) and were not different by age or parity (14).

Furthermore, there are some advantages for the insertion of Cu-IUD immediately postpartum, such as: since the cervical canal is open at the time of the delivery of the placenta, the patient experiences less discomfort from the insertion of the device than in other situations; protection against unwanted pregnancy is initiated earlier and is an opportunity, especially in low-income countries, to receive contraception as the patient is receiving medical assistance (15).

There is little information in the literature about the epidemiological profile of Brazilian women who accept or do not accept contraception in the immediate postpartum period and whether the method of choice is effective in the long run.

Aiming to contribute to a better general understanding of contraception in the immediate postpartum period, we assess the epidemiological profile of users according to the type of method chosen, and the rate of effectiveness in a municipal maternity hospital in São Bernardo do Campo.

## 2. Material and methods

### 2.1. Methodology

A cross-sectional and retrospective study at the University Hospital of São Bernardo do Campo (HMU-SBC) evaluated the electronic medical records of postpartum women enrolled from January 2016 to December 2020. The study was approved by the Hospital and ABC School of Medicine ethics committees.

We accessed all electronic medical records of postpartum women assisted at the HMU-SBC older than 18 years. The postpartum period was considered within 48 h of delivery, prior to discharge. Moreover, they were grouped as those who had inserted intrauterine cooper device intrapartum (Cu-IUD), underwent a tubal ligation during cesarean section (TL), had inserted a subdermal implant of etonogestrel (ENG), or had injected 150 mg of medroxyprogesterone acetate (DMPA). The IUD insertions occurred within the first ten minutes of delivery. Those who did not choose contraception methods were considered “no contraception method” (NCM). Cu-IUD, ENG, and DMPA information were crosscheck with the hospital pharmacy's medication dispensing control.

During hospital admission, the parturient needs to answer a standardized paper sheet containing questions about age, marital status, education degree, ethnicity, religious beliefs, and last pregnancies outcomes. In addition, cigarette, alcoholic beverages, and illicit drug use during the actual and previous pregnancy. Also, about utilization and type of contraceptive method at conception and if the pregnancy was planned, desired, or accepted. All these data and the type of delivery, information found elsewhere in the medical record, were collected.

The marital status was classified into two groups, married and stable union or single and divorced. The woman who were unmarried but co-habiting or in partnership were noted as stable union. Ethnicity was classified in Caucasian, African American, mixed (Caucasian with African American) or Asian; education degree in none, low, elementary, high school and postgraduation and religious beliefs in Catholic, Evangelical, Spiritism, Afro-Brazilian, other and no religion. Contraception use at the time of conception in “yes” or “no” and contraception type in oral or injectable contraceptive, intrauterine device, condom, or other. Cigarette, alcoholic beverages, illicit drug use, planned, desired, and accepted pregnancy were classified as “yes” or “no.”

Finally, we identified women who returned for second delivery during the study period and analyzed the contraceptive method chosen in the first hospitalization. Then the pregnancies interval and the sociodemographic characteristics were analyzed according to contraceptive method type.

### 2.2. Statistical data analysis

Numerical variables were treated as mean and standard deviation, and qualitative variables as absolute numbers and percentages. The verification of the distribution of normality was performed using the Shapiro-Wilk test. The comparison between groups with normal distribution was performed using the one-way ANOVA test, those without normal distribution Kruskal–Wallis test, and for qualitative variables Chi-square test. SPSS version 2019 software was used. Statistical tests were considered significant if the *p*-value was less than 5%.

The compared groups were constituted by the contraceptive method chosen in the immediate postpartum period. In addition, we also compared the groups with each other regarding the return rate during the study period.

## 3. Results

### 3.1. First access

As shown in Table 1, data from 20,896 women were collected, of which 8,183 (39%) had Cu-IUD inserted; 961 (4.5%) received tubal ligation; 559 (3%) chose DPMA; 204 (1%) ENG and 10,989 (52.5%) chose not to use contraception at the time of hospital discharge.

**Table 1:** Epidemiological characteristics of the population.

		IUD (*N* = 8,183)	Tubal ligation (*N* = 961)	DMPA (*N* = 559)	Implant (*N* = 204)	NCM (*N* = 10,989)	*p*
		m ± sd	m ± sd	m ± sd	m ± sd	m ± sd	
Age		28 ± 6.7^a^	35 ± 5.4^b^	26.5 ± 7.3^c^	27 ± 8^d^	29 ± 7^e^	<0.01
Number of pregnancies		2.3 ± 1.4^f^	3.8 ± 1.2^b^	2.4 ± 1.6^f^	3 ± 2.2^d^	2.2 ± 1.3^e^	<0.01
Normal birth		1.8 ± 1.2^f^	2 ± 1.2	2 ± 1.3	2.4 ± 1.8^g^	1.7 ± 1^e^	<0.01
Cesarean section		1.2 ± 0.5	2 ± 0.6^b^	1.2 ± 0.5	1.1 ± 0.4	1.2 ± 1	<0.01
Number of abortions		1.2 ± 0.6^h^	1.4 ± 0.7	1.35 ± 0.8	1.6 ± 1.3	1.3 ± 0.7	<0.01
		*N* (%)	*N* (%)	*N* (%)	*N* (%)	*N* (%)	
Type of delivery	Normal	4,940 (62.7)	15 (0.1)	358 (75)	110 (70)	5,570 (66.8)	0.01
	Cesarean section	2,929 (37.2)	892 (98)	114 (24.1)	46 (29.5)	2,763 (33.1)	
Ethnicity	Caucasian	2,795 (43)	412 (42.8)	213 (38.1)	64 (31.3)	4,756 (43.3)	0.01
	Mixed	3,273 (50.3)	485 (50.4)	304 (54.3)	111 (54.4)	5,392 (49.1)	
	Afro-American	322 (4.9)	51 (5.3)	28 (5)	13 (6.3)	551 (5)	
	Asian	9 (0.1)	2 (0.2)	2 (0.3)	1 (0.5)	31 (0.2)	
Marital status	Married-stable union	4,616 (70)	784 (81)	342 (61.1)	98 (48)	7,753 (70.6)	0.01
	Single–divorced	1,886 (29)	177 (18.4)	214 (38.4)	106 (52)	3,215 (30)	
Education degree	None	5 (0.07)	5 (0.5)	3 (0.5)	5 (2.4)	15 (0.1)	0.01
	Low	204 (3.13)	43 (4.4)	14 (2.5)	25 (12.2)	364 (3.3)	
	Elementary	1,210 (18.6)	200 (20.8)	125 (22.3)	91 (44.6)	1,908 (17.3)	
	High school	4,062 (62.4)	564 (58.6)	377 (67.4)	67 (32.8)	6,715 (61.1)	
	Postgraduation	841 (13)	123 (12.7)	34 (6)	7 (3.4)	1,594 (14.5)	
Religion	Catholic	1,948 (24)	332 (34.5)	120 (21.5)	40 (19.5)	3,303 (30)	0.01
	Evangelical	2,865 (35)	455 (47)	264 (47)	77 (38)	4,720 (43)	
	Spiritism	98 (1)	21 (2)	7 (1.5)	2 (1)	160 (1.5)	
	Afro-Brazilian	78 (1)	7 (1)	5 (1)	3 (1.5)	110 (1)	
	No religion	631 (8)	55 (6)	80 (14)	40 (19.5)	1,034 (9.5)	
	Other religions	2,563 (31)	91 (9.5)	83 (15)	42 (20)	1,648 (15)	
Contraception	Yes	2,182 (34)	368 (39)	173 (31.3)	38 (19.3)	3,419 (31.8)	0.01
	No	4,218 (66)	573 (60)	379 (68.6)	159 (80.7)	7,307 (68.1)	
Type of contraception	Oral contraception	1,478 (22.7)	220 (0.5)	111 (19.8)	17 (8.3)	2,231 (20.3)	0.01
	Injectable contraceptive	210 (3.2)	40 (4.1)	21 (3.7)	8 (3.9)	305 (2.7)	
	IUD	23 (0.3)	13 (1.3)	10 (1.7)	1 (0.4)	71 (0.6)	
	Condom	277 (4.2)	57 (6)	22 (3.9)	8 (3.9)	525 (4.7)	
	Other	4,520 (69.4)	630 (65.6)	395 (70.6)	170 (83.3)	7,860 (71.4)	
Planned pregnancy	Yes	1,804 (28.1)	216 (22.6)	151 (27.4)	28 (14)	3,722 (34.7)	0.01
	No	4,607 (71.8)	736 (77.3)	400 (72.5)	172 (86)	7,006 (65.3)	
Desired pregnancy	Yes	4,762 (75)	658 (70)	405 (74)	101 (52)	8,296 (78)	0.01
	No	1,613 (25)	281 (30)	143 (26)	94 (48)	2,332 (22)	
Accepted pregnancy	Yes	6,456 (91.5)	918 (98)	524 (96)	162 (84.8)	10,280 (97.3)	0.01
	No	598 (8.4)	18 (2)	22 (4)	29 (15.2)	283 (2.7)	
Number of pregnancies	Primiparous	2,591 (31.7)	9 (0.9)	195 (35)	63 (31)	3,994 (36.6)	0.01
	Multiparous	5,570 (68.2)	951 (99)	363 (65)	140 (69)	6,906 (63.3)	
Tobacco use	Yes	754 (11.6)	141 (14.7)	90 (16.1)	118 (57.8)	1,115 (10.1)	0.01
	No	5,751 (88.4)	820 (85.3)	469 (83.9)	86 (42.1)	9,860 (89.8)	
Alcohol use	Yes	243 (3.7)	29 (3)	34 (6)	64 (31.3)	384 (3.5)	0.01
	No	6,262 (96.2)	932 (97)	525 (94)	140 (68.7)	10,591 (96.5)	
Drug use	Yes	155 (2.4)	22 (2.2)	29 (5.1)	103 (50.4)	251 (2.3)	0.01
	No	6,350 (97.6)	939 (97.7)	530 (94.8)	101 (49.5)	10,724 (97.7)	

^a^ Bonferroni test p< 0.05 Cu-IUD group versus LT, DPMA, and absent groups.

^b^ Bonferroni test *p*< 0.05 LT group versus other groups.

^c^ Bonferroni test *p*< 0.05 DPMA group versus Cu-IUD, LT and absent groups.

^d^ Bonferroni test *p*< 0.05 IMP group versus LT and absent groups.

^e^ Bonferroni test *p*< 0.05 absent group versus other groups.

^f^ Bonferroni test *p*< 0.05 Cu-IUD and DPMA group versus LT, IMP and absent groups.

^g^ Bonferroni test *p*< 0.05 IMP group versus other groups.

^h^ Bonferroni test *p*< 0.05 Cu-IUD group versus LT and IMP groups.

When comparing the groups, those from the DPMA and ENG were younger, and those from the TL group were older (*p* < 0.05). The NCM group had the lowest number of pregnancies (2.2 ± 1.3), and the TL group had the highest (3.8 ± 1.2) when compared to the other groups (*p* < 0.05). Women referred for ENG had the highest number of vaginal births (2.4 ± 1.8), and those who underwent TL had the highest number of cesarean sections (2 ± 0.6) in comparison with the other groups (*p* < 0.05).

Of those women from the ENG group, only 48% reported having a stable family relationship or being married, while of those from TL, this number reached 81%. All groups, except for ENG, had more than 55% of women with high school completed. Women in the Cu-IUD were less catholic and protestant and showed a higher number of women with non-discriminated religions than the other groups.

For those women who inserted Cu-IUD, they were majorly mixed ethnicity (50.3%), married (70%), evangelical (35%), had high school education degree (62.4%), with no use of contraception (66%). The pregnancy was not planned (71.8%), but it was desired (75%) and accepted (91.5%). They were multiparous (68.2%) and did not made tobacco (88.4%), alcohol (96.2%) or drug (97.6%) use during the pregnancy.

The ENG group were most mixed ethnicity (54.4%), single or divorced (52%), had elementary education degree (44.6%), and were evangelical (38%), with no use of contraception (80.7%). The pregnancy was not planned (86%) and half was desired and half was not. Most of them accepted the pregnancy (84.8%). Majorly, they were multiparous (69%), used tobacco (57.8%) and drugs (50.4%) during the pregnancy, but not used alcohol (68.7%).

### 3.2. Return

As shown in Table 2, 877 women returned to the hospital for a second birth in the period analyzed. Of these, 654 (74.5%) had not opted for contraception, 31 (3.5%) for DPMA, and 189 (21.5%) had inserted Cu-IUD. Two women had undergone tubal ligation, and only one had ENG.

**Table 2:** Personal characteristics, return time and pregnancy schedule of women who returned pregnant according to the choice of method in the immediate postpartum period (IUD, DMPA or without method).

		Cu-IUD *N* = 161	DPMA *N* = 31	NCM *N* = 654	*p*
		m ± sd	m ± sd	m ± sd	
Age		27.2 ± 5,7	28.5 ± 7	28 ± 6.1	0.266
		697.1 ± 252	591.5 ± 222	692.1 ± 252	0.087
Δt. pregnancy (days)					
		*n*%	*n*%	*n*%	
Ethnicity	Caucasian	87 (46.5)	10 (32)	247 (38)	0.072
	Non-Caucasian	100 (53.5)	21 (68)	405 (62)	
Marital status	Married—stable union	133 (70.5)	21 (68)	468 (71.5)	0.867
	Single–divorced	56 (29.5)	10 (32)	186 (28.5)	
Education	Low education	13 (7)	4 (13.5)	33 (5.5)	0.094
	Elementary (second half)—High school	157 (87)	24 (80)	520 (84)	
	Higher education and postgraduate studies	10 (6)	2 (6.5)	66 (10.5)	
Religion	No religion	35 (19.5)	7 (26)	113 (19)	0.005
	Evangelical	90 (50)	16 (59.5)	279 (47)	
	Catholic	52 (29)	2 (7.5)	180 (30.5)	
	Afro-Brazilian	2 (1)	0 (0)	6 (1)	
	Spiritism	1 (0.5)	0 (0)	9 (1.5)	
	Other	0 (0)	2 (7.5)	4 (1)	
Planned pregnancy	Yes	27 (14.5)	10 (32.5)	176 (27)	0.001
	No	162 (85.5)	21 (67.5)	478 (73)	

Considering the total 20,896 women evaluated, only 2.3% from the Cu-IUD group returned, while from the DMPA group, it was 5.5% and NCM 6%. The average return time was shorter for those who opted for DMPA than the other groups. There was a higher percentage of evangelical women in the DMPA group and a lower percentage of programmed pregnancy in the Cu-IUD group.

In all of the groups, the second pregnancy was not planned, majorly. Those in Cu-IUD group were mostly non-Caucasian (53.5%), married (70.5%), had elementary or high school (87%) and were evangelical (50%). The DPMA group was composed by majority of non-Caucasian woman (68%), married (68%), with elementary or high school (80%) and evangelical religion (59.5%). The non-contraception group was most composed by non-Caucasian woman (62%), married (71.5%), with elementary or high school (84%) and evangelical religion (47%).

## 4. Discussion

It is known that >55.4% of Brazilian women who had children did not plan their pregnancy, according to a survey by the National School of Public Health of the Fundação Oswaldo Cruz (19). In this context, the possibility of providing counseling and establishing the use of effective methods makes prenatal care and hospitalization for childbirth care a window of opportunity for reproductive planning (10). In this study, many women were advised about the possibility of a method during the prenatal period, but the decision was made in the moment prior to childbirth. It is not ideal, but there is an opportunity and it should be valued in a developing country like Brazil.

In comparing the different contraception types at the early postpartum period, those who opted for the immediate insertion of a Cu-IUD were younger, prone to have a higher number of births and previous pregnancy than the group that chose not to have any method. Overall, the clinical profile between these two groups was very similar. Even those factors that showed a significant difference in clinical practice are subtle. Thus, the authors consider no specific profile of women candidates for immediate postpartum Cu-IUD insertion, and it should be offered for all women.

As Whitaker et al. (16), cited in their study, there are few restrictions on postpartum insertion of IUDs. The absolute contradiction to postpartum insertion is puerperal sepsis. We also consider other contradictions, as: rupture of membranes more than 18–24 h before delivery or chorioamnionitis prior to delivery. Despite these clinical contraindications, there are no epidemiological barriers. Furthermore, there is benefit on preventing new pregnancies in a short period of time, because the Copper-IUD promotes rapid and efficient contraception.

The epidemiological profile found in this study differs from that reported in a follow-up study of European women who had an intrauterine device (EURAS-IUD) (9). At the time of insertion, the mean age of European women was 25 ± 3.1, and the mean number of live births was 1.6; while in the population of this study, the mean was 28 ± 6.7, and the number of previous pregnancies was 2.3 ± 1.4. It should be noted that the European follow-up study evaluated women who had the IUD inserted at any time and not just postpartum. Also, we must consider whether the offer of this method is occurring at a late age in the city of São Bernardo do Campo, increasing the chance of unplanned pregnancy.

The occurrence of unplanned pregnancy was 71% and 65% in Cu-IUD and NCM groups, respectively. It is noteworthy that, in both groups, the percentage of women using some contraceptive methods was slightly higher than 30%. The oral hormonal contraceptive was the most frequent in both groups (close to 20%). Considering many women without contraceptive methods and those who use low-effectiveness methods, we can assume that the study population does not have access or adequate health equipment for reproductive planning.

The women in this study who inserted hormonal implants have specific characteristics, but different from those found in the study by Eggebroten et al. (7), they have a high average number of pregnancies and miscarriages; a higher percentage of unplanned pregnancy and most were single marital status, smokers, alcohol and drug users. The reason is that this method is only available in the hospital for women considered to be in a socially vulnerable situation.

Likewise, the group that underwent tubal sterilization has specific characteristics for being included in a protocol of legal criteria: more than two pregnancies or more than 25 years. Furthermore, all immediate postpartum TL instances are due to complications and that elective TLs were not performed in the immediate postpartum period (within 48 h of delivery). Thus, it is expected that women who underwent TL are older and have more pregnancies than other groups.

On the other hand, women assessed in Brazil have a different profile from women assessed in Ghana, by Morhe et al. (17). This study analyzed postpartum contraceptive choices among women attending a childcare clinic in Ghana and found that most of them adopted postpartum contraception with the calendar method (51.5%) and injectable contraceptives (20%) (17). This differs from our study, as a large proportion (39%) of women chose to insert a copper IUD. Like this study, most women in Ghana have had unwanted pregnancies: 22.0% wanted no more children and the last pregnancy was unintended in 44.0%. This allows us to infer that a comprehensive educational intervention is necessary to improve knowledge about family planning in both countries, even if the method choice profile is different.

For Gonie et al., acceptance of the IUD was greater in patients who had a greater number of prenatal visits (20). However, we do not know whether the issue of contraception and family planning was addressed during prenatal care.

We can also compare this study with another one carried out at Santa Casa de São Paulo from June 8 to October 8 of 2018, which analyzed the epidemiological profile of women who were offered the insertion of the copper IUD in the immediate postpartum period (18). As a result, they found that the average age of participants who accepted was 27.9 years old—similar to the age obtained in the present study (28 years old). In addition, they found that multiparity was a factor that increased IUD acceptance in the immediate postpartum period (18). This also confirms the finding in the present study, in which most of the patients who had the IUD inserted were multiparous.

In contrast, Tang et al. reported that the interest in inserting the IUD in the immediate postpartum period was lower in multiparous and older women—stating that the group preferred tubal ligation (21). However, in this study, elective TL was not available to women in the immediate postpartum period.

Of those women who underwent subcutaneous hormonal implantation on admission, less than 0.5% returned to the hospital with a second pregnancy in the period evaluated, confirming ENG with a highly effective long-term reversible method. Moreover, the return rates of women who underwent tubal ligation were also less than 0.5%, showing that this method is also effective in contraception.

The return rate of pregnant women among those who inserted Cu-IUD was 2.3%, a number consistent with the literature and lower than that presented NCM group, which had a return rate of 6%. The group that underwent DMPA had a return of 5.5%, demonstrating lower efficacy, with a rate close to NCM group. This finding supports the argument that this method should not be considered a long-term method, as it requires a user action every 3 months.

Regardless of the contraception type group, the minority of returning women programmed the pregnancy. It is noteworthy that the women who opted for DPMA in the first hospitalization had a shorter interval between births than the other groups analyzed, which had similar intervals between them. The explanation for the shorter interval between pregnancies for the DPMA group is unclear. The possibility of amenorrhea associated with an injection performed in the hospital can give a feeling of security and decrease adherence to the method. Indeed, considering the success rate and the interval between pregnancies, the indication of this method should be put into perspective for this immediate postpartum population.

## 5. Limitations

The study has limitations. The return rate may be underestimated as it is impossible to follow up with all the women or even guarantee that all who became pregnant returned to the hospital. Also, the reliability of the data depends on correctly filling out the medical records, and the retrospective methodology does not allow for an assessment of the continuity and satisfaction of the methods used. Furthermore, it is not possible to know how the patient's prenatal care went and whether this influenced the initial choice of contraceptive method.

## 6. Conclusions

According to our results and excluding women in vulnerable situations, there is no specific profile of women candidates for long-term contraceptive methods. The insertion of Cu-IUD and ENG in the postpartum period reduced the chance of returning with a new unplanned pregnancy, with a performance superior to DPMA.

The large number of women, the different types of methods evaluated with data collected over five years allow for an adequate understanding and insights into reproductive planning in the immediate childbirth context.

Thus, prenatal care and immediate postpartum should be considered moments of opportunity for reproductive planning actions, especially with the guidance and performance of LARC.

## Data Availability

The raw data supporting the conclusions of this article will be made available by the authors, without undue reservation.
